# PLK1 inhibition promotes apoptosis and DNA damage in glioma stem cells by regulating the nuclear translocation of YBX1

**DOI:** 10.1038/s41420-023-01302-7

**Published:** 2023-02-17

**Authors:** Xuetao Li, Guangliang Chen, Bin Liu, Zhennan Tao, Yue Wu, Kai Zhang, Zibin Feng, Yulun Huang, Hao Wang

**Affiliations:** 1 Department of Neurosurgery, Dushu Lake Hospital Affiliated of Soochow University, Suzhou, Jiangsu China; 2 Department of Neurosurgery & Brain and Nerve Research Laboratory, The First Affiliated Hospital of Soochow University, Suzhou, Jiangsu China; 3 Department of Neurosurgery, Qinghai Provincial People’s Hospital, Xining, Qinghai 810007 China; 4 Department of Neurosurgery, The Affiliated Drum Tower Hospital, School of Medicine, Nanjing University, Nanjing, China; 5 Institute of Soochow University, Suzhou, Jiangsu China

**Keywords:** Cancer stem cells, Targeted therapies

## Abstract

Glioma stem cells (GSCs) are the important cause of tumorigenesis, recurrence, and chemo(radio)resistance in glioma. Targeting GSCs helps improve the outcomes of glioma treatment. Polo-like kinase 1 (PLK1) is a member of the serine/threonine protein kinase family, which is highly conserved. In recent years, it has been suggested that increased levels of PLK1 and its activity are associated with tumor progression and poor prognosis. We aimed to identify whether PLK1 plays a critical role in stemness maintenance and apoptosis regulation in GSCs. Here we identify that PLK1 inhibition can induce apoptosis and DNA damage of GSCs, we have also delineat the possible underlying molecular mechanisms: PLK1 interacts with YBX1 and directly phosphorylates serine 174 and serine 176 of YBX1. Inhibition of PLK1 reduces the phosphorylation level of YBX1, and decreased phosphorylation of YBX1 prevents its nuclear translocation, thereby inducing apoptosis and DNA damage of GSCs. We confirmed that YBX1 knockdown resulted in the apoptosis and DNA damage of GSCs. These findings uncover that PLK1 inhibition induces cell apoptosis and DNA damage in GSCs through YBX1 phosphorylation, providing new insights into the mechanism by which PLK1 inhibition contributes to the apoptosis of and DNA damage in gliomas.

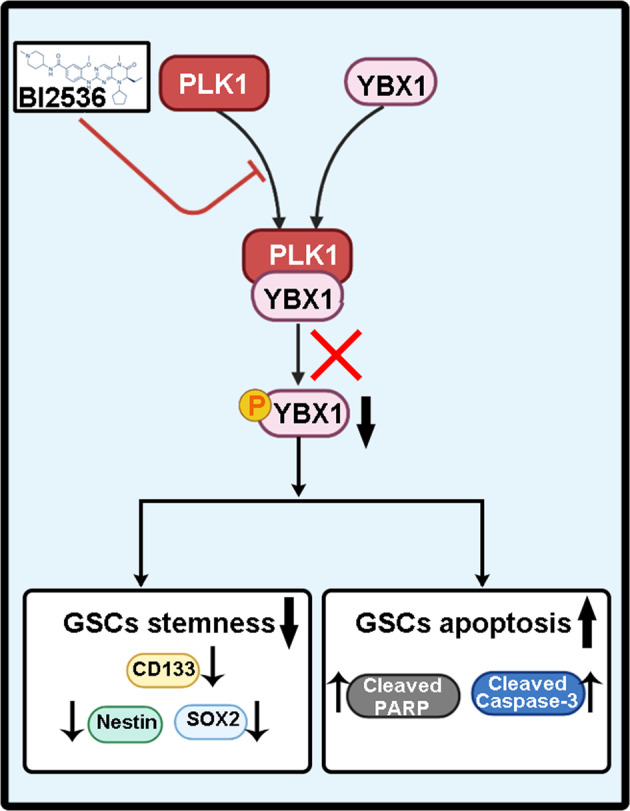

## Introduction

Glioblastoma multiforme (GBM) is the most common malignant primary brain tumor in adults that is among the most lethal of all cancers [1]. Despite the combination of surgery, radiation, chemotherapy, and immunotherapy, the 5-year survival rate is still less than 10% [1, 2]. Glioma stem cells (GSCs) are a quiescent stem-like subpopulation of glioma cells with multilineage differentiation and neurosphere formation capacity [3], and growing evidence suggests that cancer stem cells (CSCs) demonstrate significant chemoresistance and radioresistance compared with differentiated tumor cells [3–5]. Therefore, as tumor relapse drivers, these glioma stem cells must be effectively targeted to improve prognostication in GBM patients.

Polo-like kinase 1 (PLK1) is a member of the serine/threonine protein kinase family, which is highly conserved [6, 7]. PLK1 is a critical regulator of mitosis and is involved in several steps of the cell cycle, including DNA replication, centrosome maturation and separation, chromosome segregation, and cytokinesis [6–9]. Mechanistically, PLK1 directly binds to and phosphorylates different substrates in a spatiotemporal-dependent manner, thereby regulating different cellular activities. PLK1 is characterized by a C-terminal Polo-Box domain, which mediates subcellular localization and protein interactions and regulates the N-terminal serine/threonine kinase domain [10]. In recent years, the dysregulation of PLK1 leads to tumorigenesis. PLK1 is frequently upregulated in majority of human tumors including GBM but not in normal cells; furthermore, increased levels of PLK1 and its activity are associated with tumor progression and poor prognosis [7, 9–11]. Accumulating evidence implies that PLK1 inhibition can promote the apoptosis of GSCs and can suppress tumor growth [12, 13]. However, the underlying mechanism remains unclear. To clarify whether PLK1 inhibition can enhance GSC apoptosis and inhibit tumor growth, we used a proteomics-based approach to identify new phosphorylated substrates of PLK1. We ultimately identified Y-box binding protein 1 (YBX1) as a previously uncharacterized PLK1-interacting protein.

YBX1 is a member of the cold-shock domain protein family, which is encoded by the YBX1 gene. In the nucleus, YBX1 plays a pivotal role in transcription regulation; in the cytoplasm, it regulates mRNA stability and translation [14]. YBX1 is involved in several biological processes including transcription and translation regulation, mRNA precursor shearing, DNA repair, and drug resistance. Through its distinct molecular functions, Ybx1 regulates proliferation, cell differentiation, apoptosis, and cell stress response [14–16]. YBX1 is primarily distributed in the cytoplasm. On the contrary, YBX1 can be phosphorylated at several critical serine residues resulting in YBX1 translocation into the nucleus. The phosphorylation of YBX1 at S30, S34, S102, S165, and S176 promoting its nuclear translocation has been reported to date [17–19]. YBX1 is highly expressed in a variety of malignancies including GBM; in various types of cancer, YBX1 overexpression has been associated with tumor cell proliferation, invasion, apoptotic resistance, drug resistance, and poor prognosis [14, 20–22].

In this study, we demonstrated that PLK1 inhibition induces GSCs DNA damage and apoptosis both in vitro and in vivo. Treatment with a combination of PLK1 inhibitor and temozolomide (TMZ) demonstrated a synergistic effect on GSCs. Our mechanistic analysis suggested that PLK1 interacts with YBX1 and directly phosphorylates serine 174 and serine 176 of YBX1, and decreases the phosphorylation of YBX1 preventing its nuclear translocation, thereby inducing DNA damage and apoptosis of GSCs. Collectively, targeting PLK1 and combining this treatment with TMZ therapy may be novel therapeutic interventions for GBM.

## Results

### Determining the association between high expression of PLK1 and decreased survival of patients with GBM

**Fig. 1:**
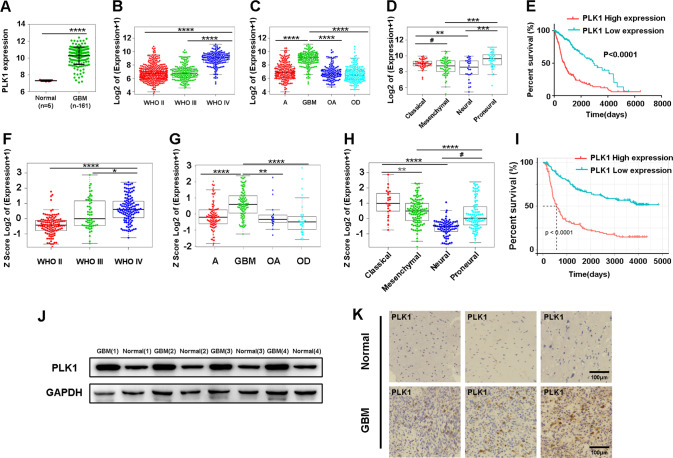
Determining the association between high expression of PLK1 and decreased survival of patients with GBM. **A** PLK1 mRNA levels from GBM tissues (*n* = 161) were significantly higher compared with those from normal tissues (*n* = 5) obtained from the TCGA database. **B**, **C** TCGA database showed PLK1 had the highest expression level in World Health Organization (WHO) grade IV glioma and GBM. **D** TCGA database showed PLK1 mRNA was higher in the proneural and classical subtypes of glioma than in the mesenchymal and neural subtypes. **E** Kaplan-Meier analysis indicated that patients with glioma with low PLK1 expression (<50th percentile) exhibited significantly improved overall survival in TCGA database (*P* < 0.0001). **F**, **G** CCGA database showed PLK1 had the highest expression level in World Health Organization (WHO) grade IV glioma and GBM. **H** CCGA database showed PLK1 mRNA was higher in the proneural and classical subtypes of glioma than in the mesenchymal and neural subtypes. **I** Kaplan-Meier analysis indicated that patients with glioma with low PLK1 expression (<50th percentile) exhibited significantly improved overall survival in TCGA database (*P* < 0.0001). **J**, **K** Protein expressions of PLK1 in glioma and normal tissues were detected by western blot and immunohistochemistry. Scale bar = 100 μm. #*P* = NS, *P < 0.5,**P < 0.1, ***P < 0.001, ****P < 0.0001, Student’s *t* test.

PLK1 is overexpressed in various types of cancers, such as prostate cancer, non-small cell lung cancer, and head and neck cancer [7, 9–11]. To assess the PLK1 expression in GBM, in silico analyses of PLK1 expression were performed using the Cancer Genome Atlas (TCGA) and the Chinese Glioma Genome Atlas (CGGA) dataset. As shown in Fig. 1A–C, results of the TCGA database analysis showed that PLK1 mRNA expression was significantly higher in the GBM tissues than in the normal tissues, and PLK1 had the highest level of expression in patients with World Health Organization (WHO) grade IV glioma and GBM. We also investigated the association between PLK1 expression and the histology of glioma in the genomic datasets. Histological subtypes included astrocytoma, GBM, oligoastrocytoma, oligodendroglioma. We found that the mRNA expression of PLK1 was higher in the proneural and classical subtypes of glioma than in the mesenchymal and neural subtypes (Fig. 1D). A Kaplan-Meier analysis was conducted to determine whether the overall survival (OS) of patients was associated with PLK1 expression in the tumors. The study patients were assigned in the high (upper 50th percentile) or low (lower 50th percentile) expression group based on the level of PLK1 expression. The Kaplan-Meier analysis indicated that patients with low PLK1 expression had better survival time than those with high PLK1 expression (Fig. 1E). Similar results were obtained in the CGGA database analysis (Fig. 1F–I). We then examined the expression of PLK1 in GBM and normal tissues by Western blot (WB) assay and immunohistochemistry (IHC). Results showed that PLK1 was highly expressed in the GBM tissues than in the normal tissues (Fig. 1J, K). These results suggested that PLK1 expression was correlated with GBM.

### PLK1 inhibition reduces the sphere formation capacity and stemness of GSCs in vitro

**Fig. 2:**
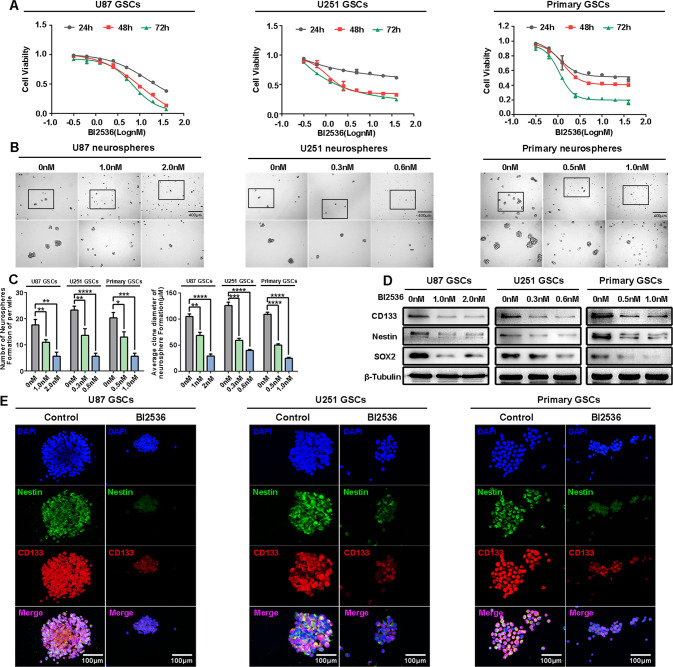
PLK1 inhibition reduces the sphere formation capacity and stemness of GSCs in vitro. **A** U87, U251, and primary GCSs were treated with BI2536 for 24, 48, and 72 h, and cell viability was detected using the Cell Counting Kit-8(CCK-8) assay. **B** The representative images of GSCs neurospheres showed that the neurosphere formation ability of GSCs was significantly inhibited by BI2536 treatment. The outlined sections of top images were defined as higher magnification sections below. *n* = 3, scale bar = 400 μm. **C** The quantification of numbers and diameter of the GSCs neurospheres shows that the neurosphere formation ability of GSCs was inhibited after BI2536 treatment. Data are shown as means ± SD, *n* = 3, #*P* = NS, *P < 0.5,**P < 0.1, ***P < 0.001, ****P < 0.0001, Student’s *t* test. **D** U87, U251, and primary GSCs were treated with BI2536 for 24 h; protein expression of CD133, Nestin, and SOX2 was detected by Western blot. **E** Cells were immunostained for Nestin (green) and CD133 (red) and stained with DAPI (blue). Scale bar = 100 μm.

In light of previous studies and bioinformatics analysis, we surmised that PLK1 may be related to GSC stemness. We also validated this hypothesis with BI2536, an ATP-competitive PLK1-specific inhibitor, which has been shown to effectively inhibit the PLK1 enzyme activity at low nanomolar concentrations [23]. First, we treated U87, U251, and primary GSCs with BI2536 for 24, 48, and 72 h, respectively, and evaluated the cellular viability by CCK8 assay to select an appropriate drug concentration (Fig. 2A). According to the IC_50_ values, the cells were all treated with BI2536 for 24 h in the subsequent experiments, and the IC_50_ concentrations were 0, 1, and 2 nM for U87 GSCs; 0, 0.3, and 0.6 nM for U251 GSCs; and 0, 0.5, and 1.0 nM for primary GSCs.

CSCs are functionally defined as tumor cells with two main characteristics: self-renewal and initiation of heterogeneous tumors that maintain the heterogeneity of the parental tumor cells [24]. Sphere formation capacity in vitro is one of the effective methods used to assess the characteristics of stem cells [24, 25]. Our results showed PLK1 inhibition in U87, U251, and primary GSCs greatly reduced the neurosphere formation (Fig. 2B). We demonstrated the effectiveness of treatment with BI2536 by measuring and quantifying the number and size of the tumor spheres from cells (Fig. 2C).

Previous findings indicated the functional differences between non-stem tumor cells and CSCs based on the enrichment of the cell surface glycoprotein CD133 [24]. Importantly, increased proportion of CD133 + cells in GBM patients was correlated with shorter OS, supporting the functional relevance of CD133 [26]. However, this single marker was not sufficient to identify the CSC population across all tumors, and a series of additional cell markers, including Nestin and SOX2, have been confirmed to have differences in their ability to self-renew and initiate tumor formation [27]. We found that PLK1 inhibition in U87, U251, and primary GSCs greatly reduced the levels of CD133, Nestin, and SOX2 (Fig. 2D, E).

Taking into account that BI2536 has weak inhibitory activity on PLK2 and PLK3, we further used shRNA to knock out the expression of PLK1 in cells, and the same results were obtained (Additional files 1). These results verified that PLK1 expression, just as its activity, is critical for GSC sphere formation and stemness maintenance.

### PLK1 inhibition can synergize TMZ-induced apoptosis and DNA damage in GSCs

**Fig. 3:**
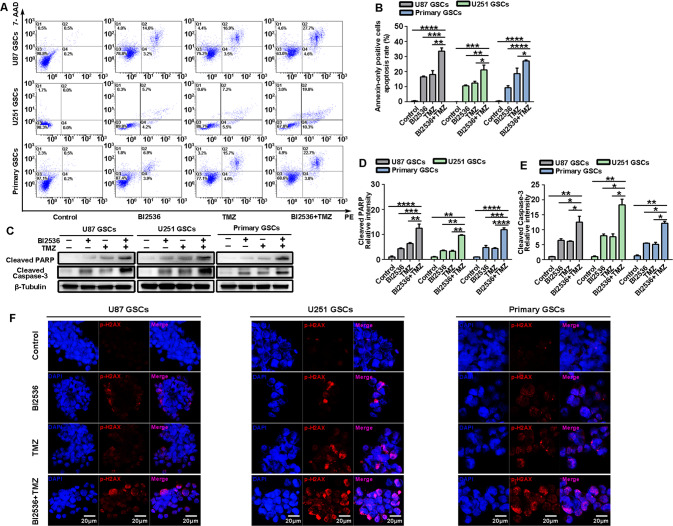
PLK1 inhibition can synergize TMZ-induced apoptosis and DNA damage in GSCs. U87, U251, and primary GSCs were treated with BI2536, TMZ, or both for 24 h. **A** Flow cytometry assay showed the apoptosis of GSCs. **B** Statistics of annexin-only positive GSCs apoptosis rate. **C**–**E** Expression of cleaved PARP and cleaved Caspase-3 was detected by Western blot. **F** Cells were immunostained for γ-H2AX (red) and DAPI (blue). Scale bar = 20 μm. Data in **B**, **D**, and **E** are shown as means ± SD, n = 3, #P = NS, *P < 0.5,**P < 0.1, ***P < 0.001, ****P < 0.0001, Student’s *t* test.

We had previously confirmed that PLK1 inhibition could induce GBM cell apoptosis [28]. Therefore, we determined whether PLK1 inhibition could induce GSC apoptosis. We then examined the apoptosis‐inducing effect of BI2536 in GSCs using the Annexin V/PE dual‐labeling tool by flow cytometry. After conducting a flow cytometry, we found that PLK1 inhibition could induce the apoptosis of U87, U251, and primary GSCs (Fig. 3A, B). Previous studies have validated the synergistic inhibition effect of BI2536 combined with TMZ on human brain GSCs in vitro and vivo [12]. In agreement with previous studies, we confirmed that the combination of BI2536 and TMZ in GSCs could synergistically induce apoptosis. Similarly, compared with a single application, cleaved poly ADP-ribose polymerase (PARP) and cleaved caspase-3 levels increased significantly after treatment with BI2536 and TMZ in all models (Fig. 3C–E).

DNA damage was considered as one of the most important cell death mechanisms induced by chemotherapy. The levels of phosphorylated H2AX on Ser139(γ-H2AX), a marker of DNA double-strand breaks, were measured by immunofluorescence confocal microscopy following BI2536, TMZ, and dual drug treatment. As shown in Fig. 3F, BI2536 or TMZ alone induced γ-H2AX in all GSC cell lines examined, and combination treatment further increased the γ-H2AX protein levels and positive cells.

Similarly, TMZ also increased the apoptotic activity and DNA damage in PLK1 knockout cells (Additional files 2), which confirmed the fact that PLK1 inhibition synergized the TMZ-induced apoptosis and DNA damage in GSCs.

### Identifying the potential direct substrates of PLK1

**Fig. 4:**
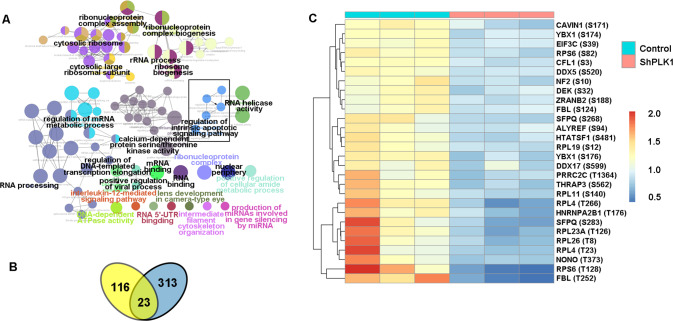
Identifying the potential direct substrates of PLK1. **A** GO analysis of proteins with significantly reduced phosphosites in PLK1 knockdown cells for biological process, molecular function, and KEGG pathway. The visualization of the results was performed with Cytoscape and ClueGOApp. **B** Venn diagram showing the number of proteins with downregulated phosphosites identified in response to PLK1 knockdown (yellow), PLK1 interaction candidates identified in the Co-IP process (blue), and overlapping proteins in the datasets. **C** The heatmap shows potential substrates of the overlapped proteins.

To identify the potential substrates of PLK1 in a systematic manner, we performed a quantitative phosphoproteomics analysis of WT and PLK1-knockout GSCs. In the liquid chromatography-tandem mass spectrometry analysis, we identified 11,974 phosphorylation sites, of which 7074 were quantified. Among them, 172 phosphorylation sites were significantly downregulated (fold change > 1.3) in the PLK1knockout cells. To gain an insight into the possible biological functions of PLK1, the protein whose phosphorylation levels were downregulated in PLK1 knockout cells were included in the bioinformatics enrichment analysis using the gene ontology and Kyoto Encyclopedia of Genes and Genomes databases utilizing the ClueGO application. In addition to the enrichment of the known PLK1 participated pathways, our analysis revealed that PLK1 could also play a role in cell apoptosis and post-transcriptional regulation (Fig. 4A). To further identify the direct substrates of PLK1 that are involved in cell apoptosis, immunoprecipitation assays, and mass spectrometry analysis was carried out to investigate the interacting partners of PLK1, as the substrates physically interact with PLK1. Finally, we obtained 313 PLK1 potentially interacting proteins. We compared the downregulated phosphorylation sites and interacting proteins from the immunoprecipitation experiment. Ideally, a true substrate of PLK1 will be identified from two orthogonal independent experiments (phosphorylated genome and interactome), in which 23 overlapping proteins (including 28 phosphorylation sites) were identified from the two experiments (Fig. 4B, C). Finally, we considered that YBX1 is a possible substrate of PLK1, because the phosphorylation level of YBX1 in PLK1 knockout cells showed significant changes and had the strongest correlation with PLK1.

### PLK1 activates YBX1 by binding with and phosphorylating YBX1

**Fig. 5:**
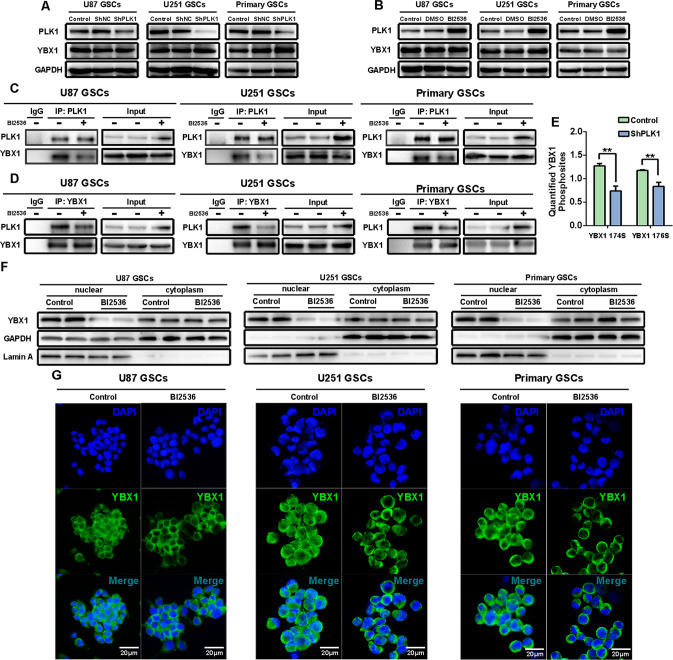
PLK1 activating YBX1 by binding with and phosphorylating YBX1. **A**, **B** U87, U251, and primary GSCs were treated with BI2536 or shPLK1 knockdown for 24 h. Expression of PLK1 and YBX1 were detected by Western blot. **C**, **D** Coimmunoprecipitation assay demonstrating that PLK1 can interact with YBX1. **E** Proteomics analysis showed PLK1 knockdown decreased phosphorylation of YBX1 at Serine174 and Serine176. Data are shown as means ± SD, *n* = 3, #*P* = NS, *P < 0.5,**P < 0.1, ***P < 0.001, ****P < 0.0001, Student’s *t* test. **F** Fractionation assays showed that PLK1 inhibition reduced the nuclear translocation of YBX1. **G** Cells were immunostained for YBX1 (green) and DAPI (blue). Scale bar = 20 μm.

Thereafter, we attempted to identify the mechanisms by which PLK1 regulated YBX1 activity. We observed that YBX1 protein expression was not affected by PLK1 in GSCs (Fig. 5A, B). As a kinase protein, PLK1 plays critical roles by phosphorylating downstream substrates. To determine whether the regulation of PLK1 on YBX1 activity is dependent on the phosphorylation of YBX1 serine/threonine sites by PLK1, co-immunoprecipitation experiments were employed. Our results revealed that endogenous PLK1 interacted with YBX1 in U87, U251, and primary GSCs (Fig. 5C, D). More importantly, our proteomics analysis showed that PLK1 knockdown decreased the phosphorylation of YBX1 at serine 174 and serine 176 (Fig. 5E). Moreover, the nuclear translocation of YBX1 is pivotal for its function, and our results showed that PLK1 inhibition reduced the nuclear translocation of YBX1 in GSCs (Fig. 5F, G). Collectively, these results indicated that PLK1 activates YBX1 by binding with and phosphorylating YBX1.

### YBX1 knockdown inducing apoptosis and DNA damage of GSCs

To evaluate the role of YBX1 in glioma, in silico analyses of YBX1 expression were performed using the TCGA and CGGA datasets. As shown in additional files 3, YBX1 mRNA was higher in glioma tissues than in normal tissues and expressed in the highest concentrations in the WHO grade IV glioma GBM. In terms of glioma subtypes, consistent with PLK1, YBX1 is highly expressed in classic and proneural subtypes. The Kaplan-Meier analysis indicated that patients with gliomas that highly expressed YBX1 (upper 50th percentile) had a shorter OS. These findings indicated that YBX1 expression negatively correlated with glioma prognosis.

**Fig. 6:**
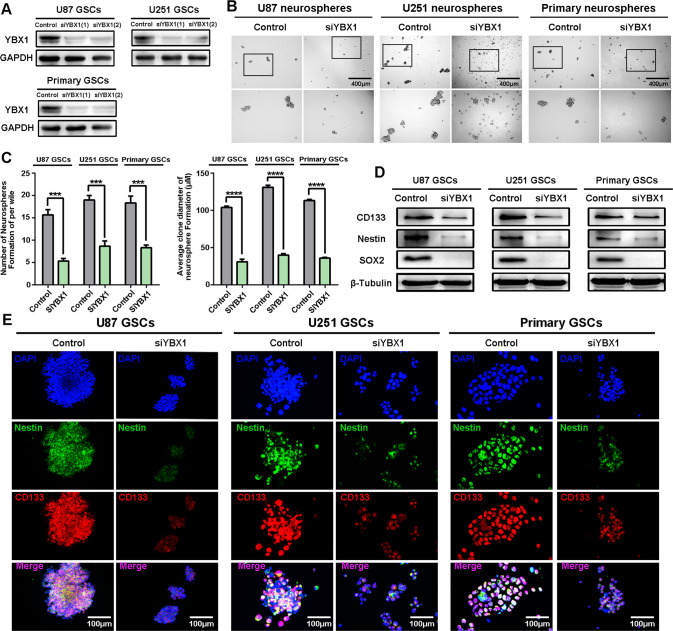
YBX1 knockdown inducing apoptosis and DNA damage of GSCs. **A** Protein expressions of YBX1 were detected by western blot. **B** The representative images of GSCs neurospheres showed that neurosphere formation ability of GSCs was significantly inhibited in YBX1 knockdown cells. The outlined sections of top images were defined as higher magnification sections below. *n* = 3, scale bar = 400 μm. **C** The quantification of numbers and diameter of the GSCs neurospheres shows that the neurosphere formation ability of GSCs was inhibited in YBX1 knockdown cells. Data are shown as means ± SD, *n* = 3, #*P* = NS, *P < 0.5,**P < 0.1, ***P < 0.001, ****P < 0.0001, Student’s *t* test. **D** U87, U251, and primary GSCs were treated with siYBX1 knockdown; protein expression of CD133, Nestin, and SOX2 was detected by Western blot. **E** Cells were immunostained for Nestin (green) and CD133 (red) and stained with DAPI (blue). Scale bar = 100 μm.

**Fig. 7:**
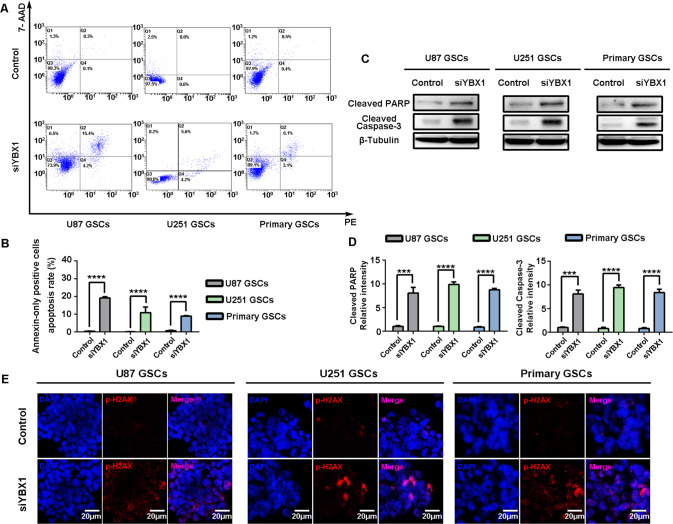
YBX1 knockdown inducing apoptosis and DNA damage of GSCs. U87, U251, and primary GSCs were treated with siYBX1 knockdown. **A** Flow cytometry assay showed the apoptosis of GSCs. **B** Statistics of annexin-only positive GSCs apoptosis rate. **C**, **D** Expression of cleaved PARP and cleaved Caspase-3 were detected by Western blot. E, Cells were immunostained for γ-H2AX (red) and DAPI (blue). Scale bar = 20 μm. Data in **B** and **D** are shown as means ± SD, *n* = 3, #*P* = NS, *P < 0.5,**P < 0.1, ***P < 0.001, ****P < 0.0001, Student’s *t* test.

In order to study the function of YBX1, we used siRNAs to knockdown the expression of YBX1 protein in GSCs. Among the two siRNAs, siRNA#1 knocked down YBX1 with the greatest efficiency and was therefore used in subsequent experiments (Fig. 6A). We found that the YBX1 knockdown GSCs gave rise to fewer and smaller spheres than the control cells (Fig. 6B, C), and the representative stem cell gene CD133, Nestin, and SOX2 were also downregulated (Fig. 6D, E). The deletion of YBX1 was previously reported to induce cell apoptosis [29]. To confirm this, we treated GSCs with siYBX1 for 24 h and detected apoptosis by flow cytometry. Our results showed YBX1 knockdown could dramatically induce GSC apoptosis (Fig. 7A, B). Additionally, we used whole cellular proteins extracted from siYBX1-treated cells to analyze apoptosis using the WB assay. YBX1 knockdown increased the expression of cleaved PARP and cleaved caspase-3, which are the hallmarks of apoptosis (Fig. 7C, D). Furthermore, siYBX1 treatment for 24 h induced dramatic phosphorylation of H2AX (γ-H2AX), indicating the occurrence of DNA damage in cells (Fig. 7E). These data demonstrated that YBX1 knockdown induced apoptosis and DNA damage in GSCs.

### Combination therapy of PLK1 inhibitor and TMZ preventing tumor growth in the xenograft glioblastoma model

**Fig. 8:**
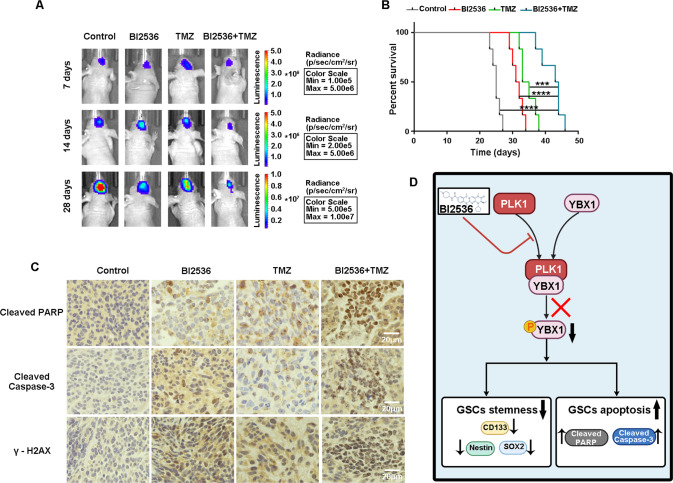
Combination therapy of PLK1 inhibitor and TMZ preventing tumor growth in the xenograft glioblastoma model. The mice were treated with intraperitoneal injection with DMSO, BI2536 (50 mg/kg/week), TMZ (20 mg/kg/day), or BI2536 (50 mg/kg/week) combined with TMZ (20 mg/kg/day) treatment. The treatment started on the 7th day after implantation and lasted for approximately 28 days. **A** Representative images of bioluminescence of mice on days 7, 14, and 28 after implantation. **B** The overall survival of mice in the DMSO, BI2536, TMZ, or BI2536 combined with TMZ treatment groups. Data are shown as the mean ± SD, *n* = 6, #*P* = NS, *P < 0.5,**P < 0.1, ***P < 0.001, ****P < 0.0001, compared to the control, ANOVA test. **C** Representative images of the IHC staining in tumor sections (×100 magnification, scale bar = 20 μm). **D** Mechanism diagram described PLK1 inhibition induces cell apoptosis and DNA damage in GSCs through YBX1 phosphorylation.

Finally, we explored the therapeutic potential of a combination of PLK1 inhibitors and chemotherapy. To generate GSC orthotopic xenograft mouse models, 2.5 × 10^5^ luciferase-labeled U87 stem cells were injected in nude mice. Tumor progression was monitored by in vivo bioluminescence imaging. Seven days after injection, vivo bioluminescence imaging suggested the success of model building (Fig. 8A). At this point, orthotopic xenograft mice were randomly assigned to the control, BI2536 (50 mg/kg/week), TMZ (20 mg/kg/day), or BI2536 (50 mg/kg/week) combined with TMZ (20 mg/kg/day) treatment group. The mice in the treatment groups were injected with the corresponding medicine, while the mice in the control group were injected with PBS intraperitoneally. Our results showed both BI2536 and TMZ could inhibit tumor growth and prolong the OS of mice, and combination therapy displayed better efficacy than a single treatment alone (Fig. 8A, B). In addition, we examined the effect of BI2536 and TMZ on the apoptosis marker and DNA damage marker levels in xenografts by IHC analysis (Fig. 8C); results showed that BI2536 or TMZ treatment increased the expression levels of cleaved PARP, cleaved caspase-3, and γ-H2AX. Collectively, these findings suggest that PLK1 inhibition exhibits potent antitumor activity in vivo, and the combination therapy of PLK1 inhibition and TMZ is an effective strategy for glioma treatment.

## Discussion

GBM is the most lethal form of glioma and is considered incurable. The median survival time of GBM patients is only 14.6 months, despite receiving new radical surgery protocols, radiotherapy, and chemotherapy with temozolomide [1, 2, 30]. The poor therapeutic efficacy and higher risk of recurrence could be partly attributed to current therapies’ inability to target GSCs, a small subpopulation of cells that are implicated in tumorigenesis, recurrence, and chemo(radio)resistance [4, 24, 31]. CSCs are functionally defined as tumor cells with two main characteristics: self-renewal and initiation of heterogeneous tumors that maintain the heterogeneity of the parental tumor cells [24]. To identify and isolate GSCs, specific and precise standards are needed. Sphere formation capacity in vitro is one of the effective methods to assess the characteristics of stem cells. Another approach to identify GSCs is to use specific surface markers to define subpopulations within the tumor that are lineage specific. The classic biomarker used to define GSCs is CD133. However, this single marker was not sufficient to identify the CSC population in all tumors, and there are several examples of CD133-CSC populations. Based on these initial studies, a series of additional cell markers, including Nestin and SOX2, that show differences in tumor initiation and self-renewal have been validated [32].

PLK1 belongs to the PLK family and plays an important role in cell cycle progression, cell proliferation, differentiation, and adaptive responses [7]. PLK1 expression is present at very low levels in most adult tissues, including the brain [33]. However, numerous studies have demonstrated that PLK1 was overexpressed in a variety of human tumors, and its high expression level often correlates with poor prognosis in cancer patients, suggesting that PLK1 is critical for tumorigenesis [7, 9–11]. To study the expression of PLK1 in glioma and its relationship with the prognosis of patients, we analyzed the TCGA and CGGA databases. Our results confirmed that the expression of PLK1 was higher in glioma tissues than in normal tissues and was negatively correlated with the patient’s prognosis. In addition, PLK1 was mainly highly expressed in classical and proneural subtypes in GBM, which may be related to the presence of tumor stem cells. Our experimental results also confirmed that PLK1 inhibition reduced the sphere formation capacity and stemness of GSCs, suggesting that PLK1 was essential for GSCs.

In recent years, PLk1 has emerged as a significant anticancer target, as its inhibition has shown remarkable antitumor effects in various cancer types. However, a complete understanding of PLK1 biology and mechanism is yet to be fully achieved. Previous studies confirmed that PLK1 controlled the FBW7 activity to induce the destruction of MYC and Myeloid cell leukemia 1 in order to regulate cell apoptosis in double-hit lymphoma [34]. Yan-Bin Feng et al. proposed that survivin is probably involved in the antiapoptotic function of PLK1 [35]. Shao et al. confirmed that modifying the phosphorylation status of Numb by Plk1 will significantly affect the cellular response to DNA damage [36]. Consistent with the reports of previous studies, our results indicated that PLK1 inhibition could induce extensive apoptosis and DNA damage of cancer cells in vivo and in vitro. To further explore the specific molecular mechanism by which PLK1 inhibition is involved in GSC apoptosis and DNA damage, we used a proteomics-based approach to identify new phosphorylated substrates of PLK1. As a result, YBX1 was identified as a new interacting protein of PLK1.

YBX1 is a member of the cold-shock domain protein superfamily, which is the most conserved nucleic acid binding protein since the evolution of bacteria and humans. YBX1 acts as both a transcription factor and a translation regulator. On the contrary, YBX1 was considered to be a transcription factor in charge of the expression of many genes through either direct or indirect interaction with the Y-box (5′-CTGATTGG-3′) or other sequences in gene promoters. By contrast, YBX1 could influence the mRNA amount not only through transcription but also through premRNA processing and mRNA stability. Previous studies have revealed that YBX1 participated in a wide variety of DNA/RNA-dependent events in two distinct modes depending on its location (either in the nucleus or in the cytoplasm), and phosphorylation of a specific serine site of YBX1 could regulate its nuclear translocation to affect the YBX1 function. In this study, we demonstrated that PLK1 promoted the YBX1 nuclear translocation by binding to YBX1 and phosphorylating its serine 174 and serine 176, which might be the mechanisms of PLK1 inhibition inducing apoptosis and DNA damage in GSCs.

YBX1 was considered to be a multifunctional oncoprotein and dysregulated a wide range of genes involved in cancer cell proliferation and survival, apoptosis, and drug resistance. In previous studies, it has been reported that YBX1 promoted the growth of cancer stem cell population and seemed to modulate the expression of numerous stemness genes, including SOX2, CD10, CD24, and CD44 [37, 38]. In addition to the regulation of tumor proliferation, YBX1 could protect cancer cells against apoptosis. A few protective mechanisms are possible. YBX1 inhibits the ability of P53 to induce cancer cell death. The inhibition of YBX1 is accompanied by the downregulation of Bcl-2, P21, P16, and cyclin D1 [39–42] and the upregulation of pro-apoptotic regulators including APAF-1, BAX, and NOXA [43]. In this study, we confirmed that the inhibition of YBX1 was related to the downregulation of the stemness genes CD133, Nestin, and SOX2, and YBX1 inhibition caused apoptosis and DNA damage of GSCs.

Finally, BI2536 abated the tumorigenicity of GSCs in intracranial xenograft models. Moreover, BI2536 and TMZ synergistically induced cell apoptosis and DNA damage in vivo, showing the potential to therapeutically target glioma cancer stem cells.

In conclusion, we demonstrated that PLK1 inhibition could induce apoptosis and DNA damage of GSCs; we have also delineated the underlying molecular mechanisms: PLK1 interacts with YBX1 and directly phosphorylates serine 174 and serine 176 of YBX1, and decreases the phosphorylation of YBX1 preventing its nuclear translocation, thereby inducing apoptosis and DNA damage of GSCs (Fig. 8D). Overall, PLK1 can be identified as a potential therapeutic target for GSCs.

## Materials and methods

### Cell culture

Glioma cells (U87 and U251) were obtained from the Shanghai Institutes for Biological Sciences. Primary cells were attained from tumor tissue of glioma patients in The First Affiliated Hospital of Soochow University. The primary cell line used in this study had been identified by Short Tandem Repeat (STR), but the relevant test results had not yet been announced. We used the following methods to sort and cultivate GSCs: Firstly, magnetically activated cell sorting (MACS) was used to collect CD133+ cells from glioma cells. Then, flow cytometer was used to quantitatively analyze the CD133+ cells in the MACS + population to test the validity of the classification. All CD133+ cells were cultured in DMEM/F-12 supplemented with B27 (Invitrogen), epidermal growth factor (10 ng/ml, R&D Systems), and basic fibroblast growth factor (5 ng/ml, R&D Systems) and were maintained in a humidified 5% CO_2_ atmosphere at 37 °C.

### Bioinformatics analysis

A normalized mRNA expression dataset for glioma was downloaded from the Cancer Genome Atlas (TCGA) and the Chinese Glioma Genome Atlas (CGGA) dataset and used to evaluate the expression of PLK1 and YBX1 transcripts. All the detailed information of glioma patients including pathology diagnosis, clinical stage, and survival data can be downloaded from the TCGA and CGGA website.

### Immunohistochemistry

The tumor tissue was taken out, fixed with paraffin, embedded, and sectioned, the section was deparaffinized in xylene, and rehydrated with a graded concentration of ethanol and distilled water. Then use 0.3% hydrogen peroxide (China) to quench the endogenous peroxidase activity, and heat the strong antigen recovery solution to 37 °C to recover the antigen. A total of 5% goat serum (Solarbio, China) was used to block non-specific proteins. The first antibody was incubated overnight at 4 °C, and the second antibody was incubated at room temperature for 60 min. Then, the slides were incubated with ABC peroxidase and diaminobenzidine (ZSGBBio). Next, the slides were counterstained with Mayer hematoxylin solution (Solarbio) for nuclear staining.

### Cell counting kit-8 (CCK8) viability assay

The viability of cells was assessed using CCK8 (Dojindo Laboratories, Kumamoto, Japan) according to the manufacturer’s protocol. GSCs were plated in 96-well plates at a density of 3 × 10^4^/ml. After the drug was added to fully react for 24, 48, 72 h, it was treated with 10 ml CCK8 at 37 °C for 2 h. Absorbance was measured at 450 nm using a microplate reader.

### Sphere formation assay

The well-grown GSCs were digested and centrifuged, then washed twice with phosphate-buffered saline (PBS), and then the cells were resuspended in a stem cell culture medium for counting. Choose an ultra-low adsorption cell culture six-well plate, add 5000 cells to each well, and then add 4 ml of medium. The culture was completed in about 2 days, and the cell count and images were taken under an inverted microscope to facilitate quantification.

### Lentiviral shRNA and small interfering RNA (siRNA) transfection

Lentiviral shRNAs against the PLK1 genes were produced by the GV112 vector (hU6-MCS-CMVPuromycin; GeneChem, China). Based on the manufacturer’s recommendation, lentiviral vectors expressing shRNA or scrambled transfected into cells. Steady cell clones transfected with shRNA-expressing constructs were chosen with puromycin intervention after infection.

The YBX1 siRNA sequence 1 was 5′-GGUUCCCACCUUACUACAU-3′ and 2 was 5′-UUCUCCGAACGUGUCACGUTT-3′. We used Lipofectamine 3000 to enhance transfection efficiency. Infection was achieved using 100 pmol siRNA and 5 μL Lipofectamine 3000 in 2 × 10^5^ cells/mL. After transfection for 24 h, we investigated the infected cells.

### Western blot

Briefly, 1 × 10^6^ cells including U87 GSCs, U251 GSCs, and primary GSCs were separately collected into microcentrifuge tubes and lysed in Western and IP lysates buffer supplied with protease inhibitors and phosphatase inhibitors on ice for 30 min and followed by high-speed centrifuge for 15 min. The cytoplasmic fraction of the supernatant was collected in another microcentrifuge tube. The cytoplasmic fraction of the supernatant was collected in another microcentrifuge tube. Protein concentration was determined by the bicinchoninic acid (BCA) kit (Beyotime, China) and balanced, and one of three proportion of loading bufffer was added for high temperature (100 °C) denaturing for 5 min. Protein was separated by 10% SDS-PAGE and transferred to PVDF membranes. The primary antibody was incubated overnight, and the second antibody was incubated at 37 °C for 1 h and captured by chemiluminescence imaging systems.

### Immunofluorescence staining

The stem cell spheres were collected and fixed with 4% paraformaldehyde. Then cells were washed three times (5 min per wash) with PBS and 0.2% Triton X-100 to permeabilize the cells at room temperature. Subsequently, we used the 5% BSA as our blocking antigen. The primary antibody was incubated overnight at 4 °C. On the next day, the cells were washed three times with PBS for 15 min each time. Then add mouse and rabbit secondary antibodies and incubate at 37 °C for 1 h. After the secondary antibody incubation, we washed the cells three times with PBS for 5 min each time, and then added 4′,6diamidino-2-phenylindole to counterstain the nuclei before observing the cells under the microscope.

### Flow cytometric analysis

Apoptosis assays were done using the Apoptosis Detection Kit (BD Biosciences, San Jose, CA, USA), according to the manufacturer’s protocol. The apoptosis and necrosis of glioma stem cells (2 × 10^5^ cells per well) were identified by PE and 7-AAD Annexin V staining. The treated cells were washed with PBS and centrifuged at 12,000 × *g* for 5 min. After suspending PE-Annexin V and 7-AAD in the binding buffer, the cells were incubated in the dark for 15 min. The results were analyzed with a FACS Calibur flow cytometer (BD Biosciences, San Jose, CA, USA).

### High-throughput phosphorylation-modified TMT label quantitative proteomics research

The protein samples were taken out from −80 °C, and 4 times the volume of lysis buffer (8 M urea, 1% protease inhibitor, 1% phosphatase inhibitor) was added, and lysed by ultrasound. Centrifuge at 12000 *g* for 10 min at 4 °C to remove cell debris, transfer the supernatant to a new centrifuge tube, and determine the protein concentration using the BCA kit. Take an equal amount of each sample protein for enzymatic hydrolysis, add an appropriate amount of standard protein, and adjust the volume to the same as the lysis solution. Slowly add 20% TCA at a final concentration, vortex to mix, and settle at 4 °C for 2 h. Centrifuge at 4500 *g* for 5 min, discard the supernatant, and wash the precipitate 2–3 times with precooled acetone. After drying the precipitate, add TEAB with a final concentration of 200 mM, ultrasonically disperse the precipitate, add trypsin at a ratio of 1:50 (protease: protein, m/m), and hydrolyze overnight. Add dithiothreitol to a final concentration of 5 mM, and reduce at 56 °C for 30 min. Then add iodoacetamide to make the final concentration 11 mM, and incubate for 15 min at room temperature in the dark. The peptides digested by trypsin were desalted with Strata X C18 (Phenomenex) and then freeze-dried in vacuo. Dissolve the peptides with 0.5 M TEAB, and label the peptides according to the instructions of the TMT kit. The peptides were fractionated by high-pH reversed-phase HPLC, and the column was Agilent 300Extend C18 (5 μm particle size, 4.6 mm inner diameter, 250 mm length). Dissolve the peptide in the enrichment buffer solution (50% acetonitrile/0.5% acetic acid), transfer the supernatant to the washed IMAC material in advance, and place it on a rotating shaker and incubate with gentle shaking. After the incubation, the material was washed three times with a buffer solution of 50% acetonitrile/0.5% acetic acid and 30% acetonitrile/0.1% trifluoroacetic acid. Finally, 10% ammonia water was used to elute the phosphopeptide, and the eluate was collected and vacuum freeze-dried. After draining, follow the instructions of C18 ZipTips for desalination, vacuum freeze draining, and then use for LC/MS analysis. The peptides were dissolved in the mobile phase A of liquid chromatography and then separated using the EASY-nLC 1200 ultra-high performance liquid system. The peptides are separated by the ultra-high performance liquid system and injected into the NSI ion source for ionization and then analyzed by HF-X mass spectrometry.

### Coimmunoprecipitation (CO-IP)

The cells were harvested and lysed using Western and IP cell lysates. Centrifuge the cell lysate at 12000 r/m for 15 min at 4 °C. After taking the supernatant to normalize the protein concentration, incubate the supernatant with protein A/G beads and primary antibodies or IgG overnight in a cold room. The beads were washed five times with Western and IP cell lysates, resuspended in sample loading buffer, and heated at 100 °C for 5 min. The supernatant was used for further western blotting.

### Intracranial xenograft model in nude mouse

The animal experiment was approved by the Animal Care Committee of the First Affiliated Hospital of Soochow University. Code of Ethics: The First Affiliated Hospital of Soochow University 2018 Code of Ethics No. 117. To evaluate whether BI2536 and TMZ inhibit tumor growth in vivo, U87 stem cells were used to establish a nude mouse model. Female BALB/c nude mice (4 weeks, 15–17 g) were purchased from the Shanghai Experimental Animal Center of the Chinese Academy of Sciences (Shanghai, China). In order to establish an intracranial model, 5 × 10^4^ U87 stem cells were stereotactically injected into mice. The mice were randomly divided into four groups with 6 mice in each group. BI2536 was dissolved in 30% PEG-400, 0.5% Tween-80, 5% propylene glycol, and TMZ was dissolved in 5% DMSO + 30% PEG300 + ddH2O. During the survival period, mice were injected intraperitoneally with PBS, BI2536 (50 mg/kg/day), TMZ (20 mg/kg/day) or BI2536 (50 mg/kg/day) combined with TMZ (20 mg/kg/day))period. Animal research is conducted in accordance with internationally recognized guidelines and national regulations. The brain was extracted and fixed in 10% formalin, and then embedded in paraffin for immunohistochemistry.

### Statistical analysis

The bar graph is represented by the mean standard deviation of at least three experimental replicates. Most experiments use the Student *t* test for statistical analysis. A one-way analysis of variance followed by Tukey’s posthoc test was used to assess the differences between the groups. The data is analyzed through the graphical keyboard 6. The significance of the *P* value is set to ^#^*P* > 0.05, *P < 0.05, **P < 0.01, ***P < 0.001, ****P < 0.0001.

## Supplementary information

Authorship change agreement

Supplementary Figure 1

Supplementary Figure 2

Supplementary Figure 3

Supplementary figure legends

Original Data File

## Data Availability

All data are available in the main text or the supplementary materials.
