# Supplementary material for: PLK1 inhibition promotes apoptosis and DNA damage in glioma stem cells by regulating the nuclear translocation of YBX1
Source: Cell Death Discov. 2023 Feb 17;9:68. doi: 10.1038/s41420-023-01302-7 (PMC9938146; doi:10.1038/s41420-023-01302-7)
Supplement: Supplementary file 1 — Authorship change agreement [file 41420_2023_1302_MOESM1_ESM.pdf]

1. Hao Wang

About the article"PLK1 inhibition promotes apoptosis and DNA damage in glioma stem cells by regulating the nuclear translocation of YBX1"

szdxwanghao@163.com 升级会员

王浩 2023-01-04 10:44
发至 szdxwanghao
详情

Yes, I agree with the changes we have made to the author list.

王浩
szdxwanghao@163.com

收起引用

---- Replied Message ----

From	王浩<szdxwanghao@163.com>
Date	1/4/2023 10:43
To	szdxwanghao@163.com<szdxwanghao@163.com>
Subject	About the article"PLK1 inhibition promotes apoptosis and DNA damage in glioma stem cells by regulating the nuclear translocation of YBX1"

Dear Hao Wang,

In the article"PLK1 inhibition promotes apoptosis and DNA damage in glioma stem cells by regulating the nuclear translocation of YBX1 (CDDISCOVERY-21-2788R1)", in order to ensure the clarity of the article after revision, we deleted the work of Xinxin Ge and Hui Yao, so we deleted the names of Xinxin Ge and Hui Yao from the article. On the other hand, we are very grateful to Bin Liu for his contribution during the revision process of the article, so Bin Liu is listed as a co-author. Do you agree with the changes we have made to the author list?

Yours sincerely,
Hao Wang

王浩
szdxwanghao@163.com

2. Xuetao Li

回复：About the article"PLK1 inhibition promotes apoptosis and DNA damage in glioma stem cells by regulating the nuclear translocation of YBX1"

szdxwanghao@163.com 升级会员

李学涛 2023-01-04 10:47
发至 szdxwanghao
详情

Yes, I agree

---- 回复的原邮件 ----

发件人	王浩<szdxwanghao@163.com>
日期	2023年01月04日 10:42
收件人	lixuetao0405@126.com<lixuetao0405@126.com>
主题	About the article"PLK1 inhibition promotes apoptosis and DNA damage in glioma stem cells by regulating the nuclear translocation of YBX1"

Dear Xuetao Li,

In the article"PLK1 inhibition promotes apoptosis and DNA damage in glioma stem cells by regulating the nuclear translocation of YBX1 (CDDISCOVERY-21-2788R1)", in order to ensure the clarity of the article after revision, we deleted the work of Xinxin Ge and Hui Yao, so we deleted the names of Xinxin Ge and Hui Yao from the article. On the other hand, we are very grateful to Bin Liu for his contribution during the revision process of the article, so Bin Liu is listed as a co-author. Do you agree with the changes we have made to the author list?

Yours sincerely,
Hao Wang

王浩
szdxwanghao@163.com

3. Guangliang Chen

Re: About the article"PLK1 inhibition promotes apoptosis and DNA damage in glioma stem cells by regulating the nuclear translocation of YBX1"

szdxwanghao@163.com 升级会员

liangliberty 2023-01-04 11:52
发至 王浩

详情

Yes, I agree

---Original---

From: "王浩"<szdxwanghao@163.com>
Date: Wed, Jan 4, 2023 10:38 AM
To: "liangliberty@163.com"<liangliberty@163.com>;
Subject: About the article"PLK1 inhibition promotes apoptosis and DNA damage in glioma stem cells by regulating the nuclear translocation of YBX1"

Dear Guangliang Chen,

In the article"PLK1 inhibition promotes apoptosis and DNA damage in glioma stem cells by regulating the nuclear translocation of YBX1 (CDDISCOVERY-21-2788R1)", in order to ensure the clarity of the article after revision, we deleted the work of Xinxin Ge and Hui Yao, so we deleted the names of Xinxin Ge and Hui Yao from the article. On the other hand, we are very grateful to Bin Liu for his contribution during the revision process of the article, so Bin Liu is listed as a co-author. Do you agree with the changes we have made to the author list?

Yours sincerely,
Hao Wang

王浩

szdxwanghao@163.com

4. Bin Liu

Re: About the article"PLK1 inhibition promotes apoptosis and DNA damagein glioma stem cells by regulating the nuclear translocation of YBX1"

升级会员

szdxwanghao@163.com

刘斌 2022-12-26 15:42
发至 王浩

详情

Yes, I agree with your decision and look forward to our next cooperation.

---Original---

From: "王浩"<szdxwanghao@163.com>
Date: Mon, Dec 26, 2022 15:39 PM
To: "25261923@qq.com"<25261923@qq.com>;
Subject: About the article"PLK1 inhibition promotes apoptosis and DNA damagein glioma stem cells by regulating the nuclear translocation of YBX1"

Dear Bin Liu,

in "PLK1 inhibition promotes apoptosis and DNA damage in glioma stem cells by regulating the nuclear translocation of In YBX1 (CDDISCOVERY-21-2788R1) "article, we are very grateful for your contribution during the revision of the article, so you are listed as the co-author. Do you agree?

Yours sincerely,
Hao Wang

王浩

szdxwanghao@163.com

5. Zhennan Tao

Re:About the article"PLK1 inhibition promotes apoptosis and DNA damage in glioma stem cells by regulating the nuclear translocation of YBX1" 精简信息

发件人: tdavid787<tdavid787@163.com>

收件人: 王浩<szdxwanghao@163.com>

时 间: 2023年01月04日 10:55 (星期三)

您的邮箱安全待提升！仅需1分钟，安全性提升30%，一键升级>>

Yes, I agree

At 2023-01-04 10:55:31, "王浩" <szdxwanghao@163.com> wrote:

- 隐藏引用文字 -

Dear Zhennan Tao,

In the article"PLK1 inhibition promotes apoptosis and DNA damage in glioma stem cells by regulating the nuclear translocation of YBX1 (CDDISCOVERY-21-2788R1)", in order to ensure the clarity of the article after revision, we deleted the work of Xinxin Ge and Hui Yao, so we deleted the names of Xinxin Ge and Hui Yao from the article. On the other hand, we are very grateful to Bin Liu for his contribution during the revision process of the article, so Bin Liu is listed as a co-author. Do you agree with the changes we have made to the author list?

Yours sincerely,
Hao Wang

王浩

szdxwanghao@163.com

6. Yue Wu

<< 返回 | 回复 | 回复全部 | 转发 | 删除 | 举报 | 拒收 | 标记为 | 移动到 | 更多

Re:About the article"PLK1 inhibition promotes apoptosis and DNA damage in glioma stem cells by regulating the nuclear translocation of YBX1" 精简信息

发件人: 吴玥<wuyue_vic@163.com>

收件人: 王浩<szdxwanghao@163.com>

时 间: 2023年01月04日 10:38 (星期三)

您的邮箱安全待提升！仅需1分钟，安全性提升30%，一键升级>>

Yes, i agree with the changes we have made to the author list.

At 2023-01-04 10:37:14, "王浩" <szdxwanghao@163.com> wrote:

- 隐藏引用文字 -

Dear Yue Wu,

In the article"PLK1 inhibition promotes apoptosis and DNA damage in glioma stem cells by regulating the nuclear translocation of YBX1 (CDDISCOVERY-21-2788R1)", in order to ensure the clarity of the article after revision, we deleted the work of Xinxin Ge and Hui Yao, so we deleted the names of Xinxin Ge and Hui Yao from the article. On the other hand, we are very grateful to Bin Liu for his contribution during the revision process of the article, so Bin Liu is listed as a co-author. Do you agree with the changes we have made to the author list?

Yours sincerely,
Hao Wang

王浩

szdxwanghao@163.com

7. Kai Zhang

Re: About the article"PLK1 inhibition promotes apoptosis and DNA damage in glioma stem cells by regulating the nuclear translocation of YBX 1"

szdxwanghao@163.com 升级会员

2289441773 2023-01-04 10:48
发至 王浩

详情

Yes, I agree.

---- Replied Message ----

From	王浩<szdxwanghao@163.com>
Date	01/04/2023 10:41
To	2289441773@qq.com<2289441773@qq.com>
Subject	About the article"PLK1 inhibition promotes apoptosis and DNA damage in glioma stem cells by regulating the nuclear translocation of YBX1"

Dear Kai Zhang,

In the article"PLK1 inhibition promotes apoptosis and DNA damage in glioma stem cells by regulating the nuclear translocation of YBX1 (CDDISCOVERY-21-2788R1)", in order to ensure the clarity of the article after revision, we deleted the work of Xinxin Ge and Hui Yao, so we deleted the names of Xinxin Ge and Hui Yao from the article. On the other hand, we are very grateful to Bin Liu for his contribution during the revision process of the article, so Bin Liu is listed as a co-author. Do you agree with the changes we have made to the author list?

Yours sincerely,
Hao Wang

王浩
szdxwanghao@163.com

8. Zibin Feng

Re: About the article"PLK1 inhibition promotes apoptosis and DNA damagein glioma stem cells by regulating the nuclear translocation of YBX 1"

szdxwanghao@163.com 升级会员

风江花月夜 2023-01-04 10:42
发至 王浩

详情

yes, I agree

---Original---

From: "王浩"<szdxwanghao@163.com>
Date: Wed, Jan 4, 2023 10:40 AM
To: "732790911@qq.com"<732790911@qq.com>;
Subject: About the article"PLK1 inhibition promotes apoptosis and DNA damagein glioma stem cells by regulating the nuclear translocation of YBX1"

Dear Zibin Feng,

In the article"PLK1 inhibition promotes apoptosis and DNA damage in glioma stem cells by regulating the nuclear translocation of YBX1 (CDDISCOVERY-21-2788R1)", in order to ensure the clarity of the article after revision, we deleted the work of Xinxin Ge and Hui Yao, so we deleted the names of Xinxin Ge and Hui Yao from the article. On the other hand, we are very grateful to Bin Liu for his contribution during the revision process of the article, so Bin Liu is listed as a co-author. Do you agree with the changes we have made to the author list?

Yours sincerely,
Hao Wang

王浩
szdxwanghao@163.com

9. Yulun Huang

<< 返回 | 回复 | 回复全部 | 转发 | 删除 | 举报 | 拒收 | 标记为 | 移动到 | 更多

Re:About the article"PLK1 inhibition promotes apoptosis and DNA damage in glioma stem cells by regulating the nuclear translocation of YBX1"

精简信息

发件人：黄煜伦<huangyulun@suda.edu.cn>
收件人：王浩<szdxwanghao@163.com>
时 间：2023年01月04日 11:00 (星期三)

翻译成中文

您的邮箱安全待提升！仅需1分钟，安全性提升30%，一键升级>>

Yes, I agree

发件人："王浩" <szdxwanghao@163.com>
发送日期：2023-01-04 11:00:27
收件人："huangyulun@suda.edu.cn" <huangyulun@suda.edu.cn>
主题：About the article"PLK1 inhibition promotes apoptosis and DNA damage in glioma stem cells by regulating the nuclear translocation of YBX1"
- 隐藏引用文字 -

Dear Yulun Huang,

In the article"PLK1 inhibition promotes apoptosis and DNA damage in glioma stem cells by regulating the nuclear translocation of YBX1（CDDISCOVERY-21-2788R1)", in order to ensure the clarity of the article after revision, we deleted the work of Xinxin Ge and Hui Yao, so we deleted the names of Xinxin Ge and Hui Yao from the article. On the other hand, we are very grateful to Bin Liu for his contribution during the revision process of the article, so Bin Liu is listed as a co-author. Do you agree with the changes we have made to the author list?

Yours sincerely,
Hao Wang

王浩
szdxwanghao@163.com
