# Supplementary figures and images for: PLK1 inhibition promotes apoptosis and DNA damage in glioma stem cells by regulating the nuclear translocation of YBX1
Source: Cell Death Discov. 2023 Feb 17;9:68. doi: 10.1038/s41420-023-01302-7 (PMC9938146; doi:10.1038/s41420-023-01302-7)

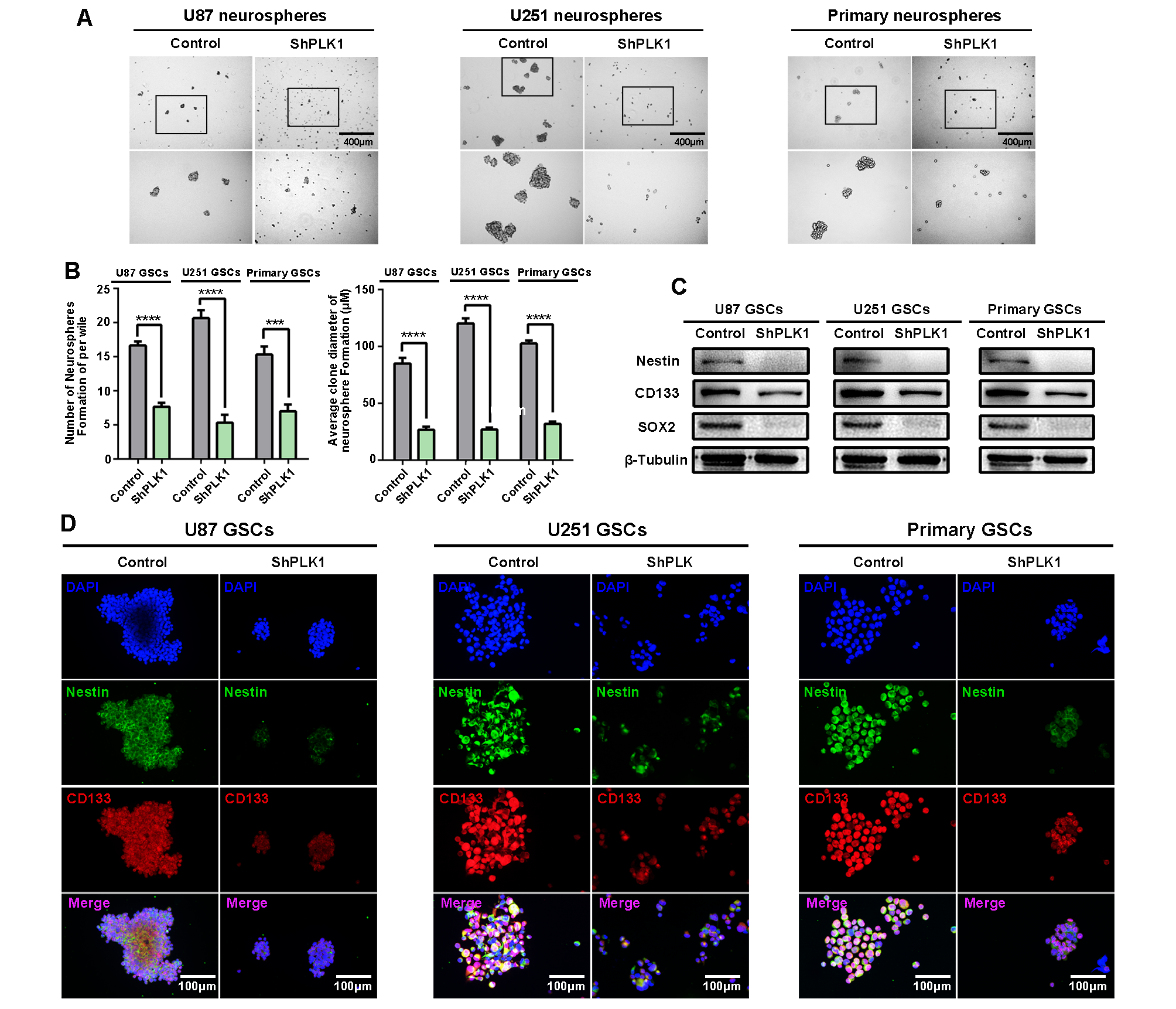

Supplement: Supplementary file 2 — Supplementary Figure 1 [file 41420_2023_1302_MOESM2_ESM.tif]

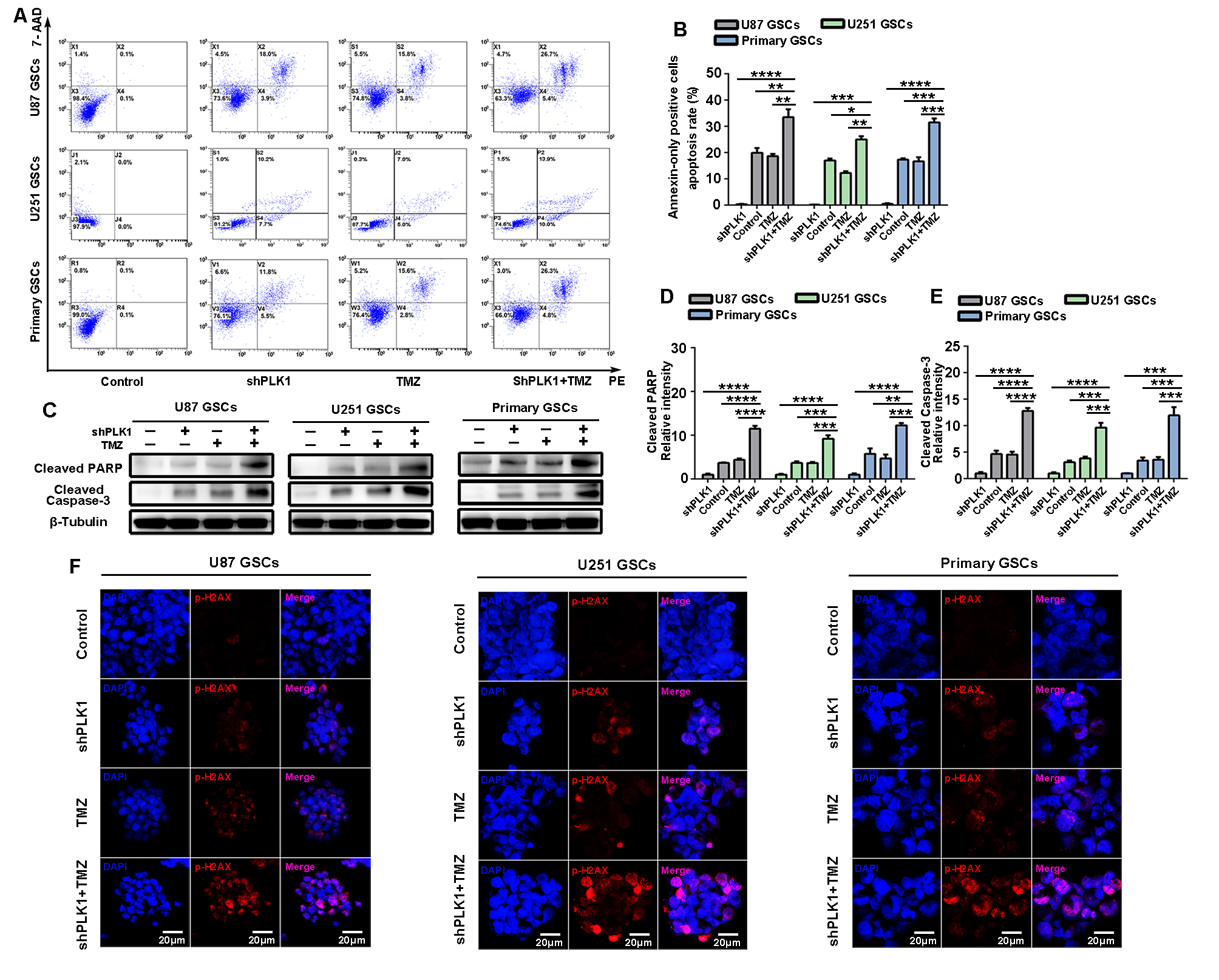

Supplement: Supplementary file 3 — Supplementary Figure 2 [file 41420_2023_1302_MOESM3_ESM.tif]

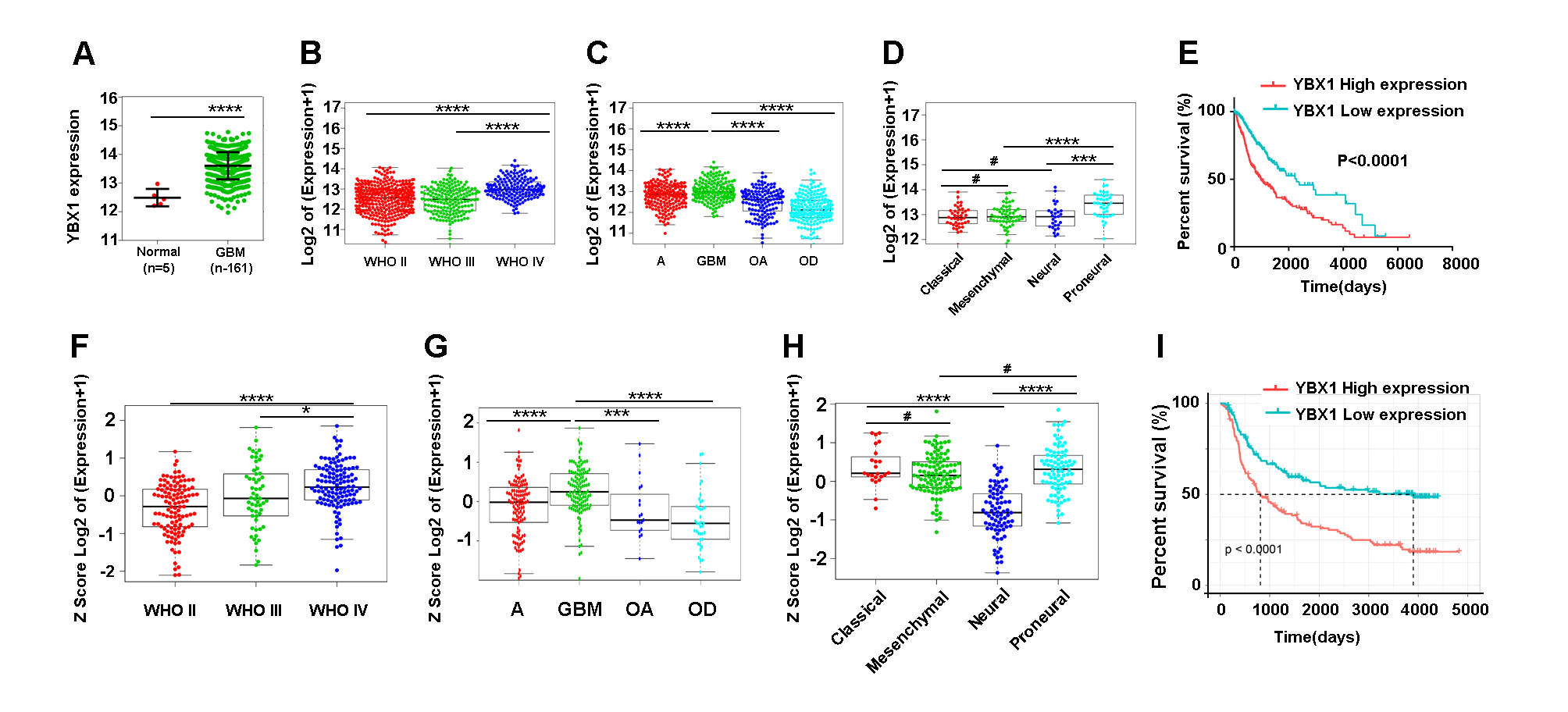

Supplement: Supplementary file 4 — Supplementary Figure 3 [file 41420_2023_1302_MOESM4_ESM.tif]

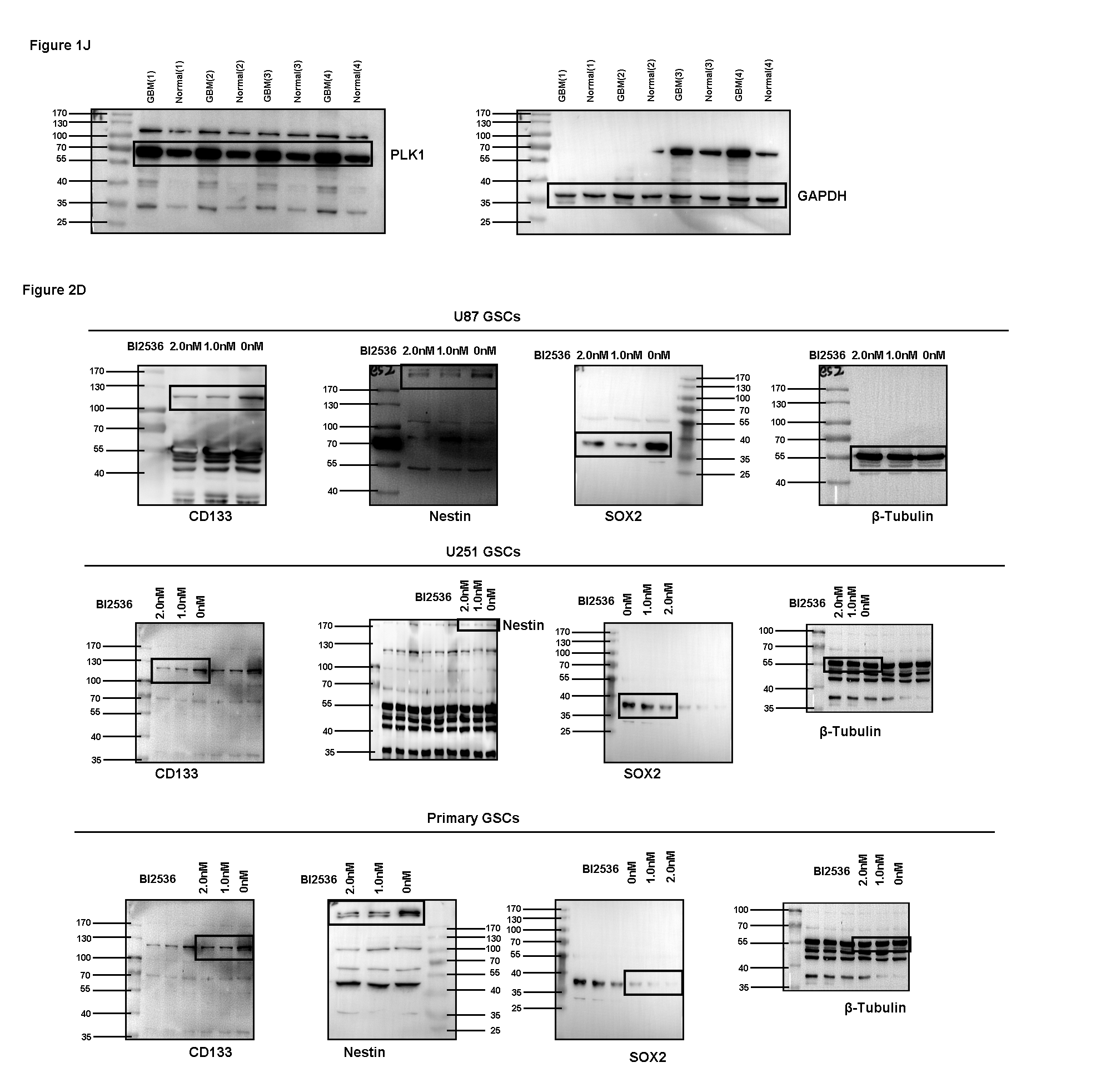

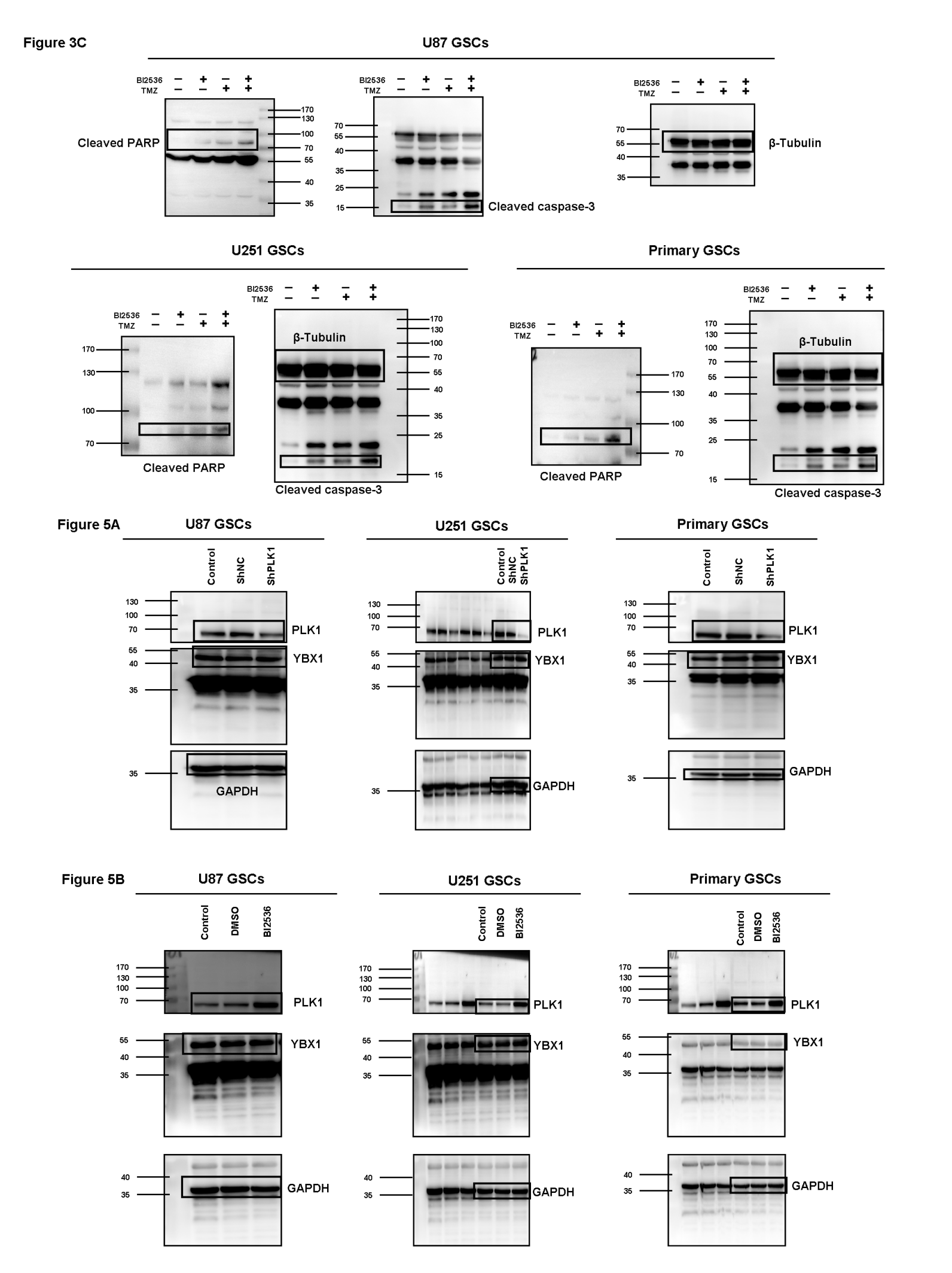

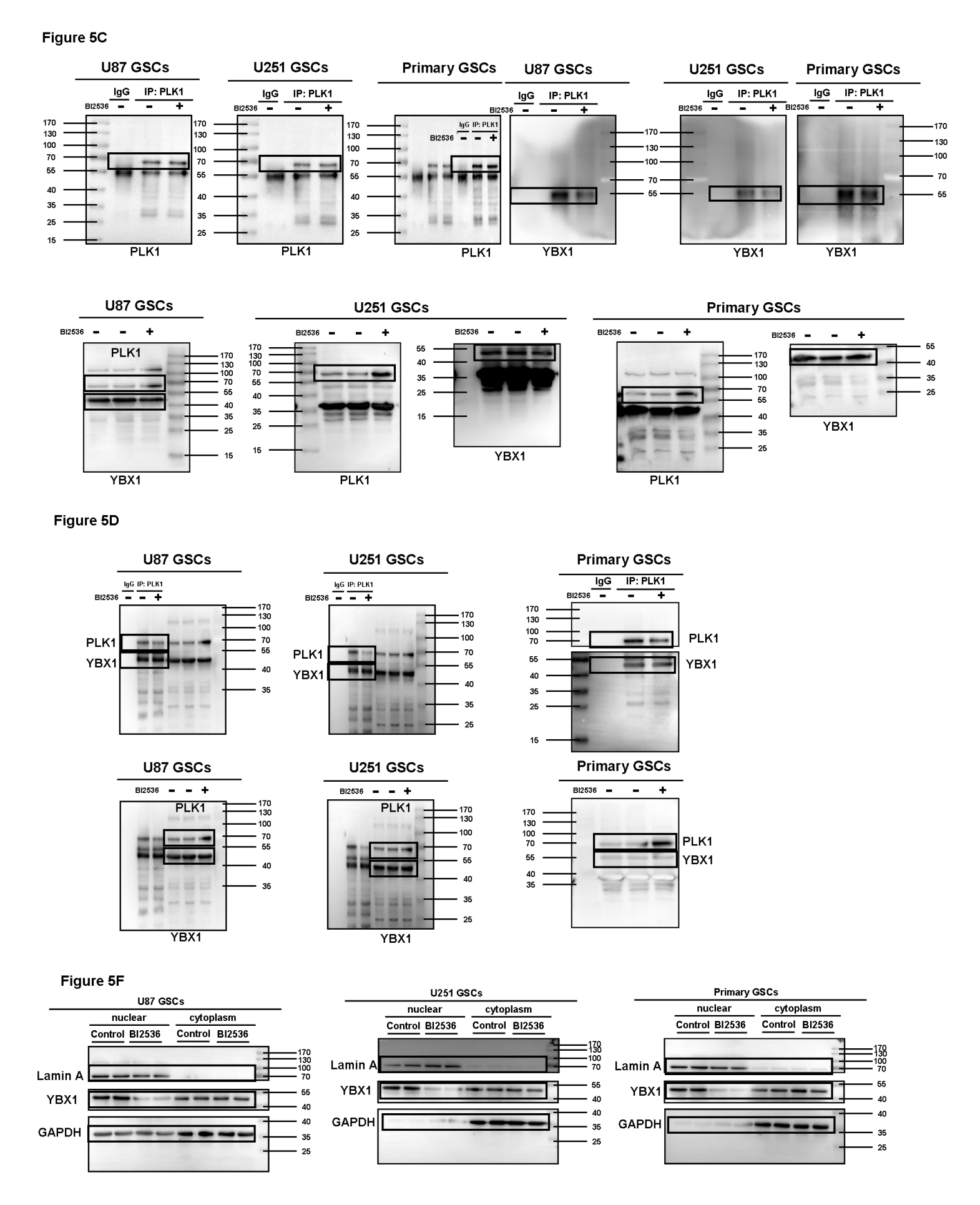

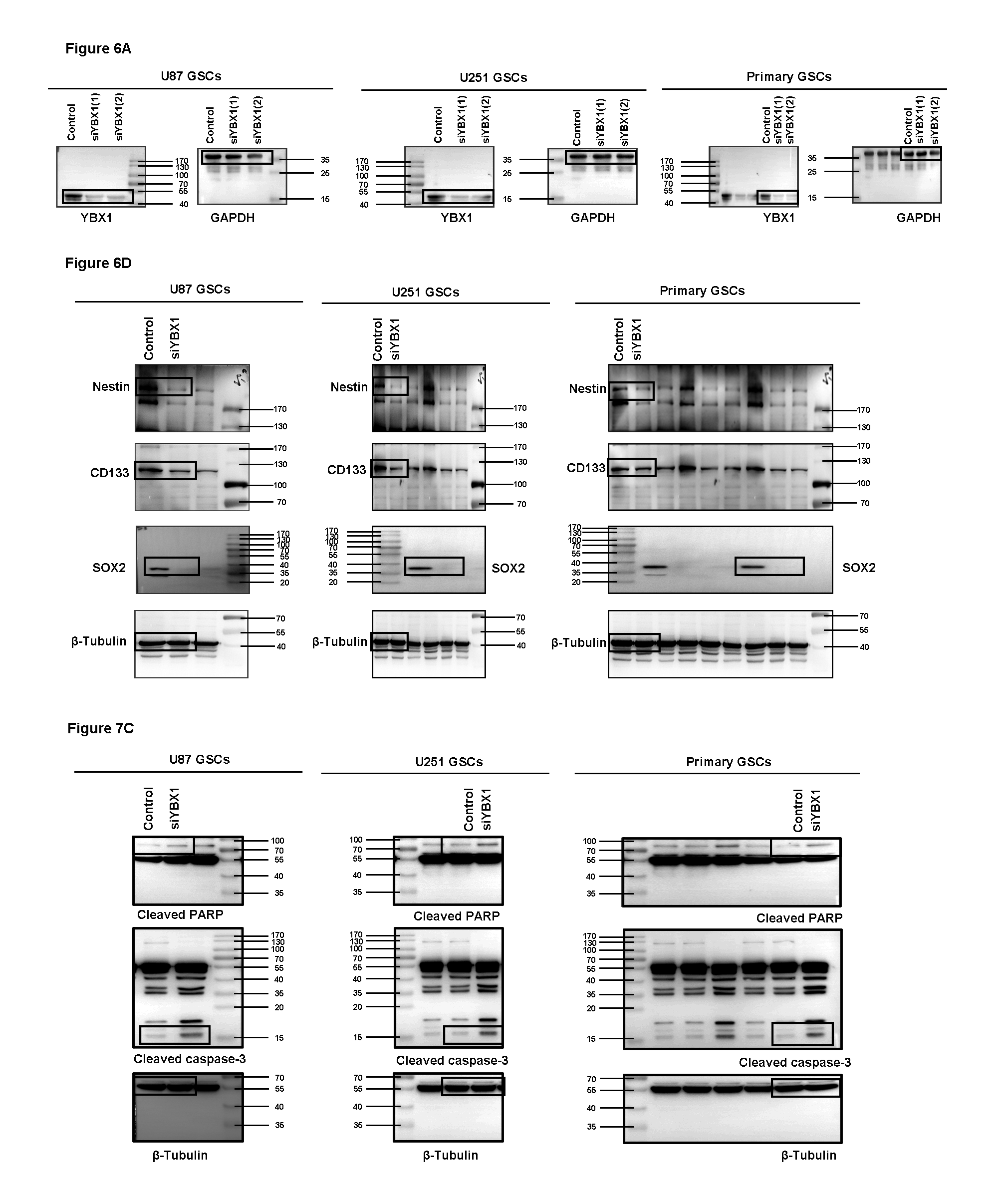

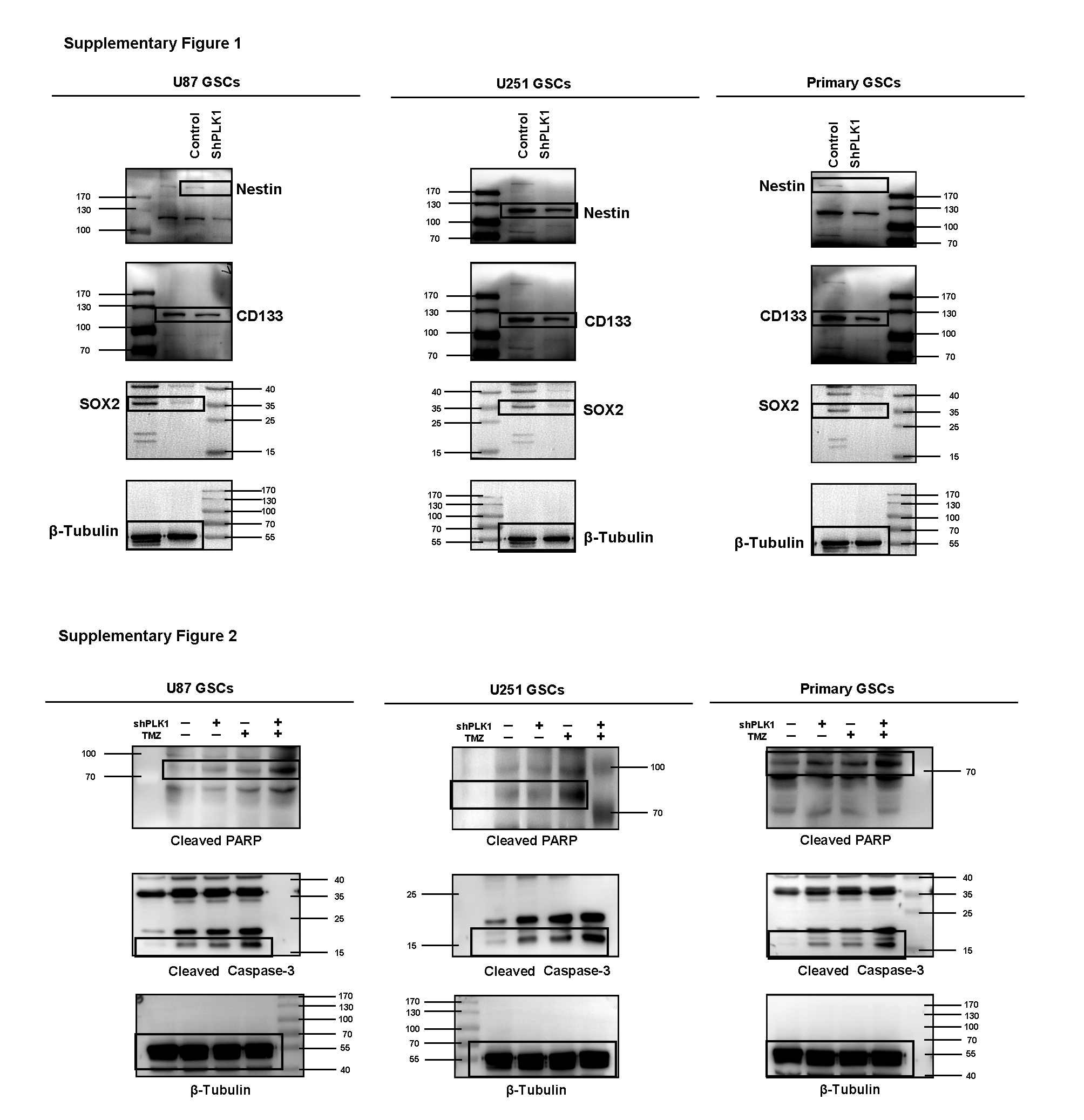

Supplement: Supplementary file 6 — Original Data File [file 41420_2023_1302_MOESM6_ESM.docx]
