# Supplementary material for: PLK1 inhibition promotes apoptosis and DNA damage in glioma stem cells by regulating the nuclear translocation of YBX1
Source: Cell Death Discov. 2023 Feb 17;9:68. doi: 10.1038/s41420-023-01302-7 (PMC9938146; doi:10.1038/s41420-023-01302-7)
Supplement: Supplementary file 5 — Supplementary figure legends [file 41420_2023_1302_MOESM5_ESM.docx]

**Supplementary Information**

**Figure S1.** PLK1 knockdown reducing the sphere formation capacity and stemness phenotype of GSCs in vitro. **A** The representative images of GSCs neurospheres showed that the neurosphere formation ability of GSCs was significantly inhibited in PLK1 knockdown cells. The outlined sections of top images were defined as higher magnification sections below. n = 3, scale bar = 400 μm. **B** The quantification of numbers and diameter of the GSCs neurospheres showing that neurosphere formation ability of GSCs was inhibited in PLK1 knockdown cells. Data are shown as means ± SD, n = 3, #P = NS, *P < 0.5,**P < 0.1, ***P < 0.001, ****P < 0.0001, Student’s t-test. **C** U87, U251, and primary GSCs were treated with shPLK1 knockdown; protein expression of CD133, Nestin, and SOX2 was detected by Western blot. **D** Cells were immunostained for Nestin (green) and CD133 (red) and stained with DAPI (blue). Scale bar = 100 μm.

**Figure S2.** PLK1 knockdown can synergize TMZ-induced apoptosis and DNA damage in GSCs. PLK1 knockdown can synergize TMZ-induced apoptosis and DNA damage in GSCs. U87, U251, and primary GSCs were treated with shPLK1 knockdown, TMZ, or both for 24h. A Flow cytometry assay showed the apoptosis of GSCs. **B** Statistics of annexin-only positive GSCs apoptosis rate. **C**-**E** Expression of cleaved PARP and cleaved Caspase-3 were detected by Western blot. **F** Cells were immunostained for γ-H2AX (red) and DAPI (blue). Scale bar = 20 μm. Data in **A**, **B** and **E** are shown as means ± SD, n = 3, #P = NS, *P < 0.5,**P < 0.1, ***P < 0.001, ****P < 0.0001, Student’s t-test.

**Figure S3.** High expression of YBX1 is associated with decreased GBM survival. High expression of YBX1 is associated with decreased GBM survival. **A** YBX1 mRNA levels from GBM tissues (n = 161) were significantly higher compared with those from normal tissues (n = 5) obtained from the TCGA database. **B**, **C** TCGA database showed YBX1 had the highest expression level in World Health Organization (WHO) grade IV glioma and GBM. **D** TCGA database showed YBX1 mRNA was higher in the proneural and classical subtypes of glioma than in the mesenchymal and neural subtypes. **E** Kaplan-Meier analysis indicated that patients with glioma with low YBX1 expression (<50th percentile) exhibited significantly improved overall survival in TCGA database (P<0.0001). **F**, **G** CCGA database showed YBX1 had the highest expression level in World Health Organization (WHO) grade IV glioma and GBM. **H** CCGA database showed YBX1 mRNA was higher in the proneural and classical subtypes of glioma than in the mesenchymal and neural subtypes. **I** Kaplan-Meier analysis indicated that patients with glioma with low YBX1 expression (<50th percentile) exhibited significantly improved overall survival in TCGA database (P<0.0001). #P = NS, *P < 0.5,**P < 0.1, ***P < 0.001, ****P < 0.0001, Student’s t-test.
